# Spatial navigation in young versus older adults

**DOI:** 10.3389/fnagi.2013.00094

**Published:** 2013-12-19

**Authors:** Ivana Gazova, Jan Laczó, Eva Rubinova, Ivana Mokrisova, Eva Hyncicova, Ross Andel, Martin Vyhnalek, Katerina Sheardova, Elizabeth J. Coulson, Jakub Hort

**Affiliations:** ^1^Memory Clinic, Department of Neurology, 2nd Faculty of Medicine, Charles University in Prague and Motol University HospitalPrague, Czech Republic; ^2^International Clinical Research Center, St. Anne’s University Hospital BrnoBrno, Czech Republic; ^3^School of Aging Studies, University of South FloridaTampa, FL, USA; ^4^Queensland Brain Institute, The University of QueenslandBrisbane, QLD, Australia

**Keywords:** spatial navigation, aging, allocentric navigation, egocentric navigation, spatial learning, gender, Alzheimer’s disease, hippocampus

## Abstract

Older age is associated with changes in the brain, including the medial temporal lobe, which may result in mild spatial navigation deficits, especially in allocentric navigation. The aim of the study was to characterize the profile of real-space allocentric (world-centered, hippocampus-dependent) and egocentric (body-centered, parietal lobe dependent) navigation and learning in young vs. older adults, and to assess a possible influence of gender. We recruited healthy participants without cognitive deficits on standard neuropsychological testing, white matter lesions or pronounced hippocampal atrophy: 24 young participants (18–26 years old) and 44 older participants stratified as participants 60–70 years old (*n* = 24) and participants 71–84 years old (*n* = 20). All underwent spatial navigation testing in the real-space human analog of the Morris Water Maze, which has the advantage of assessing separately allocentric and egocentric navigation and learning. Of the eight consecutive trials, trials 2–8 were used to reduce bias by a rebound effect (more dramatic changes in performance between trials 1 and 2 relative to subsequent trials). The participants who were 71–84 years old (*p* < 0.001), but not those 60–70 years old, showed deficits in allocentric navigation compared to the young participants. There were no differences in egocentric navigation. All three groups showed spatial learning effect (*p*’ s ≤ 0.01). There were no gender differences in spatial navigation and learning. Linear regression limited to older participants showed linear (β = 0.30, *p* = 0.045) and quadratic (β = 0.30, *p* = 0.046) effect of age on allocentric navigation. There was no effect of age on egocentric navigation. These results demonstrate that navigation deficits in older age may be limited to allocentric navigation, whereas egocentric navigation and learning may remain preserved. This specific pattern of spatial navigation impairment may help differentiate normal aging from prodromal Alzheimer’s disease.

## INTRODUCTION

Aging involves accumulation of adverse biological, psychological, and social changes over time (Bowen and Atwood, 2004) that may or may not signal pathology. Because of the long preclinical period of Alzheimer’s disease (AD), recognizing normal and pathological aging has been challenging and the frontier between these two conditions is blurred (Sperling et al., 2011). The relatively high prevalence of AD makes this an important public health issue. Age-related changes interfere unevenly with cognitive functioning (Gazova et al., 2012). While certain cognitive domains do show a decline, other may remain stable (Burke and Barnes, 2006).

Navigation in space is a complex cognitive function that is essential for independence, safety, and quality of life. Differences in spatial navigation between young and older adults were demonstrated by previous research (Barrash, 1994; Wilkniss et al., 1997; Burns, 1999; Newman and Kaszniak, 2000; Moffat and Resnick, 2002; Driscoll et al., 2005; Iaria et al., 2009; Head and Isom, 2010; Jansen et al., 2010). The decline in spatial navigation was shown to be apparent after 60 years of age and further accelerated after 70 years of age (Barrash, 1994). Studies performed in virtual reality showed a specific pattern of spatial navigation deficits in older adults restricted to allocentric navigation (Moffat and Resnick, 2002; Iaria et al., 2009). Allocentric navigation is world-centered processing of spatial information, when individuals have to rely on a “spatial map” using distant landmarks. It was shown to be dependent on medial temporal lobe structures, especially the hippocampus (Grön et al., 2000; Moffat et al., 2006). According to functional neuroimaging studies, reduced hippocampal activation occurs during spatial navigation tasks in older adults compared to their young counterparts (Moffat et al., 2006; Antonova et al., 2009). Therefore, hippocampal dysfunction may be responsible for any allocentric deficits in older adults. Egocentric, or body-centered, spatial navigation where distance and directions from individuals’ body position are used for navigation, is instead parietal lobe dependent (Maguire et al., 1998) and was shown not to be affected in older adults (Rodgers et al., 2012).

However, studies in real-space environment testing separately allocentric and egocentric navigation in older adults are lacking. General spatial navigation learning seems to be unimpaired in older age according to some studies (Barrash, 1994; Newman and Kaszniak, 2000). However, specific comparison of allocentric and egocentric navigation in the real-space setting has not yet been reported. Due to specific age-related changes in spatial navigation, older individuals may avoid new environments and become restricted to well-known familiar places.

Further, there is evidence suggesting that the ability of spatial navigation and spatial learning is severely impaired in patients with AD and contributes to the loss of functional independence. This impairment is present very early in the course of AD, even in pre-dementia stages with the same pattern as in the clinical dementia stage (Mapstone et al., 2003; deIpolyi et al., 2007; Hort et al., 2007; Laczó et al., 2009, 2011, 2012), where atrophy of the hippocampus (Nedelska et al., 2012) and parietal cortex (Weniger et al., 2011), known biomarkers for AD, is the likely culprit. However, differentiation between age-related spatial navigation changes and spatial navigation impairment in the very early, preclinical, stage of AD may be challenging. Furthermore, the situation is complicated by white matter (WM) lesions that are commonly present in the brain of AD patients and also cognitively normal elderly people and may influence spatial navigation performance (Weniger et al., 2011).

Although much work has been done in the field of age-related spatial navigation changes, some issues still remain unsolved. Recent studies showing spatial navigation deficits in older adults were performed in the virtual reality settings that lack vestibular and proprioceptive feedback and therefore may not fully reflect navigation in the real world. On the other hand, original studies investigating spatial navigation in older adults that were performed in the real-space settings did not discriminate between allocentric and egocentric spatial navigation and learning.

Further, findings of spatial navigation changes in the older adults may be biased when using an unselected cohort of older patients defined as normal only on the basis of neuropsychological test results. Because WM lesions and hippocampal atrophy suggestive of preclinical stage of AD may impair spatial navigation, it is desirable to exclude participants with these pathologies to get a more homogeneous cohort of healthy and cognitively normal older adults. Beside age, gender may also influence spatial navigation as indicated by previous research, where men outperformed women in several spatial navigation tasks (Moffat et al., 1998; Astur et al., 1998; Saucier et al., 2002; Chai and Jacobs, 2009; Woolley et al., 2010), especially in allocentric navigation (Saucier et al., 2002), where a possible explanation may lie in a different activation of the left hippocampus in men and women (Grön et al., 2000). However, a recent study performed in a real-world setting reported no gender differences in spatial navigation (Burke et al., 2012). Although research exploring the link between gender and spatial navigation has been extensive in the past 20 years, the majority of studies were performed in virtual reality settings with young participants, and thus studies conducted in the real-space environment separating allocentric and egocentric navigation and focused on elderly are still lacking.

Using the real-space human analog of the Morris Water Maze (hMWM) that allows for separate testing of two basic spatial navigation strategies and using a selected cohort of older adults without pronounced hippocampal atrophy (indicative of incipient AD) or WM lesions that may affect spatial navigation performance, we assessed the differences between young and older adults and possible influence of gender on real-space allocentric and egocentric spatial navigation and learning.

Specifically, the first aim of this study was to characterize the profile of spatial navigation performance and learning in young versus older adults. The older adults were further stratified based on previous spatial navigation research (Barrash, 1994) into participants 60–70 years old and those 71–84 years old, all of whom were free of WM lesions or pronounced hippocampal atrophy to reflect genuine physiological spatial navigation deficit in older age. We hypothesized that in older adults spatial navigation performance would be worse compared to young adults, mainly in allocentric navigation. The second aim was to evaluate the influence of gender on the real-space navigation performance and learning irrespective of age, given that female gender was also reported to interfere with allocentric navigation (Astur et al., 1998; Saucier et al., 2002). The third aim was to assess whether allocentric and egocentric navigation performance would decline in a linear or curvilinear (quadratic) fashion in participants 60 years of age and older.

## MATERIALS AND METHODS

### PARTICIPANTS

Older adult participants (60–84 years, *n* = 62) without memory complaints, neurological and psychiatric disorders and psychiatric medication were recruited from the seniors attending University of the Third Age at Charles University in Prague or from relatives of patients of the Memory Clinic, Motol University Hospital in Prague. Young adult participants (18–26 years, *n* = 24) were mostly students of medicine or psychology and were selected to be matched to elderly participants by sex and education. All subjects underwent standard medical and neurological examination, complex neuropsychological and spatial navigation testing. Subjects with memory complaints, history of neurological or psychiatric disease, psychiatric medication, abnormal neurological examination including gait or movement difficulties, were not included. Elderly subjects further underwent magnetic resonance imaging (MRI) brain scan.

Participants meeting DSM IV-TR criteria for dementia (*n* = 1), Petersen’s criteria for mild cognitive impairment (Petersen, 2004) (*n* = 3) or scoring more than 1.5 SD below the age- and education-adjusted norms on neuropsychological examination (*n* = 7) were excluded. Seven more participants were excluded due to abnormal images of the brain (see Magnetic resonance imaging for details).

Therefore, the final sample included 68 participants: 24 young participants 18–26 years old and 44 older participants were included in the analyses. The older adult participants were further stratified into two subgroups–participants 60–70 years old (*n* = 24) and participants 71–84 years old (*n* = 20). This stratification was adopted from a study by Barrash (1994) in which apparent changes in spatial navigation were observed after age 60 and even greater changes after age 70. Similar stratification was used in some neuropsychological studies (e.g., Whelihan and Lesher, 1985). Finally, this stratification corresponds to neuropsychological findings suggesting that decline in cognitive domains such as executive function, working memory, and long-term memory becomes empirically observable after 60 years of age (Treitz et al., 2007; Park et al., 2002), and working memory decline appears further accelerated after 70 years of age (Park et al., 2002).

All participants involved in this study had signed written informed consent that was approved by a local ethics committee.

### NEUROPSYCHOLOGICAL TESTING

Comprehensive neuropsychological battery that was used to assess all cognitive domains of participants consisted of Auditory Verbal Learning Test, Free and Cued Selective Reminding Test, Logical Memory II, Brief Visuospatial Memory Test – Revised, Rey–Osterrieth Complex Figure Test (Copy and Recall Condition), Clock Drawing Test, Digit Span Task (Forward and Backward), Digit Symbol–Coding Test, Stroop test (Victoria version), Trail Making Test (A and B), Controlled Oral Word Association Test, Semantic Fluency Test, Boston Naming Test. Mini-Mental State Examination was used to evaluate global cognitive functions.

### MAGNETIC RESONANCE IMAGING

Magnetic resonance imaging was performed using a 1.5T MRI scanner (Gyroscan; Philips Medical Systems, The Netherlands). Scans were inspected by a neuroradiologist to ensure appropriate data quality. Two participants with relevant brain pathology (meningioma) were excluded. Visual scoring was performed to evaluate hippocampal atrophy (Scheltens et al., 1992) and WM lesions (Fazekas et al., 1991) on a MRI brain scan. WM lesions were evaluated using Fazekas scale (Fazekas et al., 1991) on axial sections of T2-weighted and FLAIR sequences. Fazekas scale is a 4-point visual scale (0–3), where “0” signifies absence of WM lesions, “1” signifies sporadic WM lesions, “2” signifies confluence of WM lesions, and “3” signifies severe WM lesions. Subjects with moderate to severe WM lesions – Fazekas score ≥2 points were excluded (*n* = 2). Hippocampal atrophy was evaluated using Scheltens visual scale (Scheltens et al., 1992) on coronal sections of T1-weighted 3D FFE sequences. Scheltens visual scale is a 5-point medial temporal lobe atrophy (MTA) rating scale (0–4), where grades are assessed according to width of temporal horn, length of chorioidal fissure, and preservation of height of hippocampus, with “0” signifying no atrophy and “4” signifying the most severe atrophy. The MTA scores were assessed for the right and left side of the brain separately. The images were evaluated by two experienced raters blinded to the clinical diagnosis and results of neuropsychological and spatial navigation tests. A definite score was assigned when consensus was reached. Subjects with hippocampal atrophy – MTA score above the age-adjusted cut-offs (Scheltens et al., 1992) – ≥2 on any side in subjects ≤75 years (*n* = 1) and ≥3 in subjects >75 years (*n* = 1) were excluded. One subject with simultaneous WM lesions and hippocampal atrophy was also excluded.

### SPATIAL NAVIGATION TESTING

**FIGURE 1:**
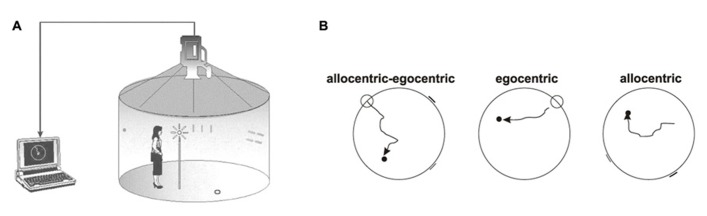
**Human analog of the Morris Water Maze**. **(A)** In-scale diagram of the real-space navigation setting. **(B)** The scheme of three individual subtasks: allocentric–egocentric, egocentric, and allocentric (courtesy of K. Vlček).

Spatial navigation tests were performed in the Laboratory of Spatial Cognition in the Department of Neurology, 2nd Faculty of Medicine, Charles University in Prague, Czech Republic, a joint workplace with Institute of Physiology Academy of Sciences of the Czech Republic v.v.i., Prague, Czech Republic. The hMWM is designed to separately test two basic types of navigation–allocentric and egocentric. Allocentric (world-centered) navigation, hippocampus-dependent, that is independent of an individual’s position and where salient distal cues (landmarks) are used for navigation (Astur et al., 2002). Egocentric (body-centered) navigation is considered parietal cortex-dependent, and relies on an individual’s position and the start location (Maguire et al., 1998). The participants were tested in the real-space version of the hMWM that was located in the navigation setting called the Blue Velvet Arena – a fully enclosed cylindrical arena 2.8 m in diameter surrounded by a 2.9 m high dark blue velvet curtain (**Figure 1A**). The design of the Blue Velvet Arena and the real-space testing procedure were described in detail elsewhere (Laczó et al., 2009; Laczó et al., 2010). The aim was to locate the invisible goal in three different subtasks using the start position or two distal orientation cues, respectively (**Figure 1B**).

The allocentric–egocentric subtask was a training task to make the subject familiar with the test and involved locating the goal using its spatial relationship with both the start position and the two distal orientation cues. The egocentric subtask involved using only the start position to locate the goal with no distal orientation cues displayed. The allocentric subtask involved using only two distal orientation cues at the perimeter for navigation to the goal as the start position was unrelated to the goal position. Each subtask involved eight trials. The relative positions of the goal, start position, and both orientation cues were identical across all trials. The correct position of the goal as well as its relationship to the start position and to the orientation cues was shown after each trial in each subtask to facilitate learning. The performance was measured as the distance error between the subject’s final position and the actual goal location (in centimeters). There was no time limit to find the goal, mainly to reduce bias by differences in cognitive, sensory, and physical functioning.

### STATISTICAL ANALYSIS

An analysis of variance (ANOVA) with *post hoc* Tukey’s test of honestly significant differences (HSD) evaluated mean differences between the groups in gender, years of education, and neuropsychological measures. A χ^2^ test evaluated differences in proportions (gender). The distance between the participant’s final position and the correct goal location (distance error) measured in centimeters was used in the analyses as the measure of navigational accuracy (dependent variable), whereas group status was the independent variable. These main analyses included the assessment of between-group and between-gender differences in spatial navigation performance and learning effects in the egocentric and allocentric subtasks separately. We used a repeated measures (RM) ANOVA with two between-subjects factors (group: young versus young–old versus old–old and gender: female versus male) and one within-subjects factor (trial: trials 2–8). Note that trial 1 was not used in the analyses to reduce possible bias by a rebound effect, whereby the performance changes more dramatically between the first and second trial relative to subsequent trials. Again, *post hoc* Tukey’s test was used to compare individual groups.

Linear regression was used to evaluate age-related differences in spatial navigation in participants 60–84 years old, where spatial navigation accuracy was the dependent variable and age (linear effect) and age × age (quadratic effect) were the independent variables.

Statistical significance was set at two-tailed (alpha) of 0.05. All analyses were conducted by using SPSS for Windows.

## RESULTS

The groups did not differ in gender and education (*p’*s > 0.05). The descriptive comparisons regarding demographic characteristics and neuropsychological measures are displayed in the **Table 1**.

**Table 1:** Characteristics of the Sample by Age Group.

Variables	Participants 18–26 years old	Participants 60–70 years old	Participants 71–84 years old
Age, mean (SD), years	22.45 (4.9)	67.74 (5.6)	75.50 (5.8)	
Education, mean (SD), years	15.55 (0.6)	14.84 (0.5)	16.19 (0.6)	
Women, No (%)	15 (62.5)	17 (70.8)	13 (65.0)	
Mini-Mental State Examination, mean (SD)	29.73 (0.5)	29.16 (1.4)	28.31 (1.2)^**^	
Geriatric Depression Scale, mean (SD)	1.36 (1.8)	2.32 (3.5)	2.00 (2.2)	
Auditory Verbal Learning Test 1–5, mean (SD)	60.75 (6.5)	50.95 (9.413)	41.56 (7.394)^***^	
Auditory Verbal Learning Test 30, mean (SD)	13.18 (1.6)	10.58 (3.0)^*^	8.50 (2.6)^***^^†^	
Free and Cued Selective Reminding Test – free recall, mean (SD)	10.18 (0.8)	9.84 (0.4)	10.19 (0.5)	
Free and Cued Selective Reminding Test – total recall, mean (SD)	15.82 (0.4)	15.95 (0.2)	15.94 (0.3)	
FAS Verbal Fluency Test, mean (SD)	40.36 (11.0)	47.11 (10.7)	42.94 (11.1)	
Trail Making Test A, mean (SD)	30.55 (5.7)	35.56 (15.0)	38.96 (8.8)	
Trail Making Test B, mean (SD)	64.55 (19.0)	78.63 (25.2)	105.06 (23.5)^***^^††^	
Digit Span Forward Task – points, mean (SD)	10.00 (2.8)	10.21 (2.2)	8.31 (1.9)^*^^†^	
Digit Span Backward Task – points, mean (SD)	8.45 (2.3)	7.26 (1.6)	5.50 (2.3)^***^^†^	
Rey Osterrieth Complex Figure Test – recall condition, mean (SD)	26.18 (5.2)	17.61 (3.9)^***^	16.13 (5.1)^***^	
Egocentric Navigation Test, mean (SD), cm	18.88 (1.0)	26.35 (3.6)	27.27 (3.7)	
Allocentric Navigation Test, mean (SD), cm	22.86 (2.0)	31.41 (2.7)	41.80 (4.9)^***^	

* *p* < 0.05

** *p* < 0.01

*** *p* < 0.001 compared to participants 18–26 years old.

† *p* < 0.05

†† *p* < 0.01 compared to participants 60–70 years old. SD, standard deviation; cm, centimeters.

**FIGURE 2:**
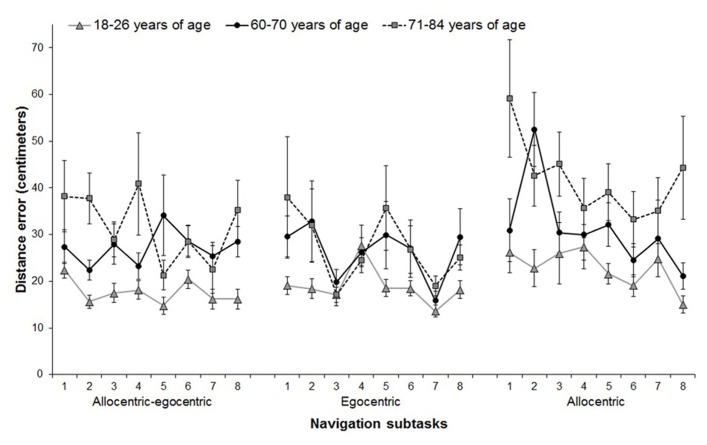
**Performance across individual trials in three spatial navigation subtasks for the three age groups**. Mean distance errors from the goal with SD are depicted for each trial. Trial 1 was excluded from the analyses to reduce possible bias by a rebound effect. Allocentric–egocentric subtask was not included in statistical analyses, because it was intended as a learning trial to familiarize participants with testing procedure. In the allocentric subtask, the participants 71–84 years old made on average significantly more distance errors than those 18–26 and 60–70 years of age. No significant results were observed for the egocentric subtask. All groups improved their performance in a similar way across seven consecutive trials.

In the main analyses, we first addressed our first hypothesis that spatial navigation performance would be impaired in older participants. We found a significant main effect for group performance in the allocentric subtask (*F*[2,64] = 9.40; *p* < 0.001), where the participants 71–84 years old consistently exhibited poorer overall spatial navigation accuracy than the participants 60–70 years old (*p* < 0.001; **Figure 2**). There were no differences in the allocentric navigation accuracy between the young participants and those 60–70 years old (*p* = 0.182). Differences between the participants 60–70 years old and those 71–84 years old were significant (*p* = .043). The main effect for group performance in the egocentric subtask was not significant (*F*[2,64] = 1.74; *p* = 0.184) indicating no differences in egocentric navigation across groups. However, the resultant performance was not due to failure to execute the task as a learning effect, based on a change in performance across consecutive trials in the sample overall, was observed for all groups in the allocentric (*F*[6,384] = 2.72, *p* = 0.022) and the egocentric (*F*[6,384] = 3.50, *p* = 0.020) subtasks. There were no significant group-by-trial interactions, suggesting no differences in learning among the groups in the allocentric (*F*[12,384] = 1.50; *p* = 0.140) and egocentric (*F*[12,384] = 0.99; *p* = 0.429) subtasks.

We next addressed the second hypothesis, that gender would influence spatial navigation performance. We did not find any main effect for gender in the allocentric (*F*[2,64] = 0.08; *p* = 0.777) and egocentric (*F*[2,64] = 0.15; *p* = 0.704) subtasks. Further, there were no significant gender-by-trial interactions, suggesting there were no gender differences in learning in the allocentric (*F*[6,384] = 1.18; *p* = 0.319) or egocentric (*F*[6,384] = 0.50; *p* = 0.664) subtasks. There were also no significant gender-by-group-by-trial interactions, suggesting no gender differences in learning among the groups in the allocentric (*F*[6,384] = 0.51; *p* = 0.484) and egocentric (*F*[6,384] = 0.332; *p* = 0.906) subtasks.

Finally, linear regression analyses were used to address the third hypothesis regarding whether greater error distance on allocentric and egocentric spatial navigation tasks would be associated with age in participants 60 years of age and older, and whether the decline would be linear or quadratic. We found that scores in allocentric navigation performance did get progressively worse for the older participants (standardized regression coefficient [β] = 0.30, *p* = 0.045). We also found a quadratic effect (β = 0.30, *p* = 0.046), indicating that worsening of spatial navigation performance was further accelerated in older ages. There was no linear (β = 0.06, *p* = 0.722) or quadratic (β = 0.06, *p* = 0.713) effect of age on egocentric navigation.

## DISCUSSION

We used a real-space hMWM to investigate the differences in spatial navigation performance between young and older participants and to assess the influence of gender on spatial navigation and learning. We compared young participants (18–26 years old) with two groups of cognitively normal older participants: participants 60–70 years old and those 71–84 years old who did not present with WM lesions or pronounced hippocampal atrophy. Consistent with our hypotheses, we found spatial navigation deficits in allocentric navigation in participants 71–84 years old. There were no significant differences between young and older participants in egocentric navigation. Both allocentric and egocentric spatial learning was preserved in older participants compared to young participants. Further, we found that gender did not influence spatial navigation or learning in the real-space environment. Finally, we found that worsening of allocentric navigation with age was gradual, with further acceleration in older ages.

Our results are consistent with previous studies describing general spatial navigation deficits in older adults compared to their younger counterparts (Barrash, 1994; Wilkniss et al., 1997; Burns, 1999; Newman and Kaszniak, 2000; Moffat and Resnick, 2002; Driscoll et al., 2005; Iaria et al., 2009; Head and Isom, 2010; Jansen et al., 2010) and later studies in virtual reality showing selective allocentric navigation impairment (Moffat and Resnick, 2002; Iaria et al., 2009) accompanied by a compensatory shift from hippocampus-dependent (allocentric) to non-hippocampal (egocentric) strategy (Rodgers et al., 2012).

From the clinical point of view, it is important to be able to differentiate between physiological spatial navigation deficit in older age and spatial navigation impairment in prodromal or even preclinical stages of AD. These differences may lie in a different pattern and quantity of spatial navigation impairment (Mapstone et al., 2003; deIpolyi et al., 2007; Hort et al., 2007; Laczó et al., 2009, 2010, 2011, 2012). Specifically, even very early in the course of AD, besides profound allocentric navigation impairment, egocentric navigation is also affected, presumably due to atrophy of parietal cortex, especially precuneus (Weniger et al., 2011). However, differentiation between age- and AD-related spatial navigation changes, especially in the preclinical stage of AD remains challenging.

In our study cognitively normal participants demonstrated spatial learning effect (by presenting improvement across seven consecutive trials in allocentric and egocentric navigation) compared to patients in the early stage of AD, where spatial learning was found to be impaired (Hort et al., 2007; Laczó et al., 2009, 2011, 2012). Thus, spatial learning does not seem to be influenced by age in cognitively normal adults, differentiating them from patients with early stage AD where pronounced hippocampal atrophy (Nedelska et al., 2012), accumulation of pathological tau (Braak and Braak, 1991) and beta amyloid proteins are present in the brain.

We did not find any effect of gender on allocentric or egocentric spatial navigation performance and learning. Our results are in concordance with current literature showing that male and female participants can learn spatial tasks equally well (Astur et al., 1998; Moffat et al., 1998; Saucier et al., 2002; Chai and Jacobs, 2009; Woolley et al., 2010). However, spatial navigation performance and navigation strategies were found to be gender dependent, with men showing an advantage over women (Astur et al., 1998; Moffat et al., 1998). Specifically, women tended to make more errors relative to men in use of the allocentric navigation (Saucier et al., 2002). A possible cause of gender differences in spatial navigation was may be different levels of activation of the left hippocampus and the right parietal and prefrontal cortex between men and women (Grön et al., 2000). However, all studies reporting superiority of males in spatial navigation were conducted with young participants and decreased levels of testosterone are associated with worse spatial navigation (Driscoll et al., 2005). Thus our findings suggesting no relation between gender and spatial navigation performance may be caused partially by recruitment of older cohort in which hormonal differences are less pronounced.

Furthermore, the previously reported effects of gender on spatial navigation in young participants was observed only in the virtual reality setting (Astur et al., 1998; Moffat et al., 1998; Saucier et al., 2002; Chai and Jacobs, 2009; Woolley et al., 2010) and a recent study performed in a real-world setting reported no between-gender differences in spatial navigation (Burke et al., 2012), similar to our findings. More studies are thus needed to solve the issue of gender influence on spatial navigation in the real-world setting.

One strength of our study is the use of the real-space hMWM, which allows for separate evaluation of two basic navigation strategies (allocentric and egocentric) and spatial learning effect. The real-space setting mimics very well navigation in the real world due to vestibular and proprioceptive feedback that contributes to successful navigation. Further cognitively normal older participants were precisely selected to be free of WM lesions and pronounced hippocampal atrophy that were found to affect spatial navigation performance (Weniger et al., 2011; Nedelska et al., 2012). In the absence of WM lesions and pronounced hippocampal atrophy in our older adult sample, we speculate that allocentric navigation deficits in participants 71–84 years of age may be a result of reduced hippocampal activation in response to a spatial navigation task, as previously demonstrated by functional neuroimaging studies (Moffat et al., 2006; Antonova et al., 2009).

Some limitations of this study should be mentioned. Due to the lack of availability of participants 27–59 years old we were not able to assess age-related changes in spatial navigation through the entirety of the life course. However, it is possible that we still captured most of the age-related differences in spatial navigation as previous research suggests that decline in cognitive domains such as executive function, working memory, and long-term memory may become apparent only after 60 years of age (Park et al., 2002; Treitz et al., 2007). Still, a future study with participants representing all decades of adult life should be conducted. Additional limitation is the use of a cross-sectional design, which makes it impossible to evaluate longitudinal changes. Therefore, we are not able to fully exclude the possibility of future development of cognitive impairment eventually leading to dementia despite the current absence of hippocampal atrophy or WM lesions. Future research that adopts a longitudinal design may be needed.

## CONCLUSION

In summary, our results suggest that, in cognitively healthy older adults, spatial navigation deficit in the real-space environment may be limited to allocentric navigation. Egocentric spatial navigation and learning appear to be preserved in older age. This specific pattern of spatial navigation impairment may help differentiate normal aging from prodromal AD.

## Conflict of Interest Statement

Dr. Laczó has consulted for Pfizer and holds shares of Polyhymnia-TS*. Dr. Hort has consulted for Pfizer, Janssen, Merck, Novartis, Elan, Zentiva, Ipsen and holds shares of Polyhymnia-TS*. Other co-authors declare that they have no commercial or financial relationships that could be construed as a potential conflict of interest. (*Polyhymnia-TS also conducts research in spatial navigation in its own facility using the same paradigm as was used in this study, although the setting itself differs from that used in this study. Polyhymnia-TS has no influence on research presented by our research group, which includes research presented in this manuscript.)
